# ZnI_2_-Mediated *cis*-Glycosylations of Various Constrained Glycosyl Donors: Recent Advances in *cis*-Selective Glycosylations

**DOI:** 10.3390/molecules29194710

**Published:** 2024-10-04

**Authors:** Akihiro Ishiwata, Xuemei Zhong, Katsunori Tanaka, Yukishige Ito, Feiqing Ding

**Affiliations:** 1 RIKEN Cluster for Pioneering Research, Wako 351-0198, Japan; kotzenori@riken.jp (K.T.); yukito@chem.sci.osaka-u.ac.jp (Y.I.); 2 School of Pharmaceutical Sciences (Shenzhen), Shenzhen Campus of Sun Yat-sen University, Shenzhen 518107, China; zhongxm36@mail2.sysu.edu.cn; 3 Medical College, Shaoguan University, Shaoguan 512026, China; 4 Department of Chemical Science and Engineering, Tokyo Institute of Technology, Tokyo 152-8552, Japan; 5 Graduate School of Science, Osaka University, Toyonaka 560-0043, Japan

**Keywords:** 1,2-*cis*-glycoside, stereoselective *cis*-glycosylation, oligosaccharides, ZnI_2_, constrained glycosyl donors

## Abstract

An efficient and versatile glycosylation methodology is crucial for the systematic synthesis of oligosaccharides and glycoconjugates. A direct intermolecular and an indirect intramolecular methodology have been developed, and the former can be applied to the synthesis of medium-to-long-chain glycans like that of nucleotides and peptides. The development of a generally applicable approach for the stereoselective construction of glycosidic bonds remains a major challenge, especially for the synthesis of 1,2-*cis* glycosides such as β-mannosides, β-L-rhamnosides, and β-D-arabinofuranosides with equatorial glycosidic bonds as well as α-D-glucosides with axial ones. This review introduces the direct formation of *cis*-glycosides using ZnI_2_-mediated *cis*-glycosylations of various constrained glycosyl donors, as well as the recent advances in the development of stereoselective *cis*-glycosylations.

## 1. Introduction

**Figure 1:**
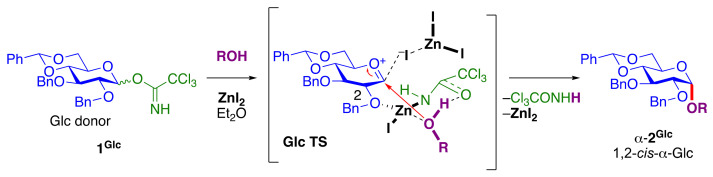
ZnI_2_-mediated 1,2-*cis*-α-D-glucopyranosylation. TS was obtained by DFT calculations (Section 2.8.1).

Stereoselective 1,2-*cis O*-glycosylation is one of the most essential issues in synthetic carbohydrate chemistry for the construction of various glycans with biological functions [1,2,3,4,5,6,7,8,9]. The preparation of 1,2-*trans O*-glycoside became possible using a stereoselective glycosylation method based on the effect of neighboring group participation from acyl carbonyl functionality at the 2-position of the glycosyl donor [10,11,12,13,14,15,16,17,18]. By the activation of the glycosyl donor, the kinetically favored *cis*-participation of the acyl group at the 2-position of the donor to the anomeric carbon at the 1-position occurs, followed by the nucleophilic attack of the acceptor from the opposite side to afford the 1,2-*trans O*-glycoside stereoselectively. Compared to the 1,2-*trans O*-glycosylations, the highly stereoselective synthesis of 1,2-*cis* glycosides is far less straightforward. The stereochemical outcome of a chemical glycosylation reaction is influenced by multiple chemical and environmental factors, including the structure of the glycosyl donor, the type and position of protecting groups installed on the donor, the nucleophilicity of the acceptor, the solvent in which the reaction is performed, the concentration of substrates, and the reaction temperature, and is determined by the specific combination of these factors [19,20,21,22,23,24,25,26,27,28,29]. The 1,2-*cis*-configured *O*-glycosidic linkages, such as α-glucopyranoside, β-mannopyranoside, β-L-rhamnopyranoside, β-D-arabinofuranosides, and 2-azido-2-deoxy-α-D-glucopyranoside, are found in natural glycans, especially in glycoconjugates (glycoproteins, glycolipids, proteoglycans, and microbial polysaccharides) and glycoside natural products [30,31,32,33,34,35]. Chemical glycosylation is a useful method to obtain these glycosidic linkages as the alternative way of isolation from natural sources. However, the strictly controlled formation of these 1,2-*cis* glycosides is generally difficult, and the key factors controlling the stereoselectivity of glycosylation are not fully understood. This review introduces a direct formation of *cis* glycosides using recently developed ZnI_2_-mediated *cis* glycosylations of various constrained glycosyl donors [36,37,38,39,40,41] (Figure 1).

### Recent Development of Stereoselective cis Glycosylations

**Figure 2:**
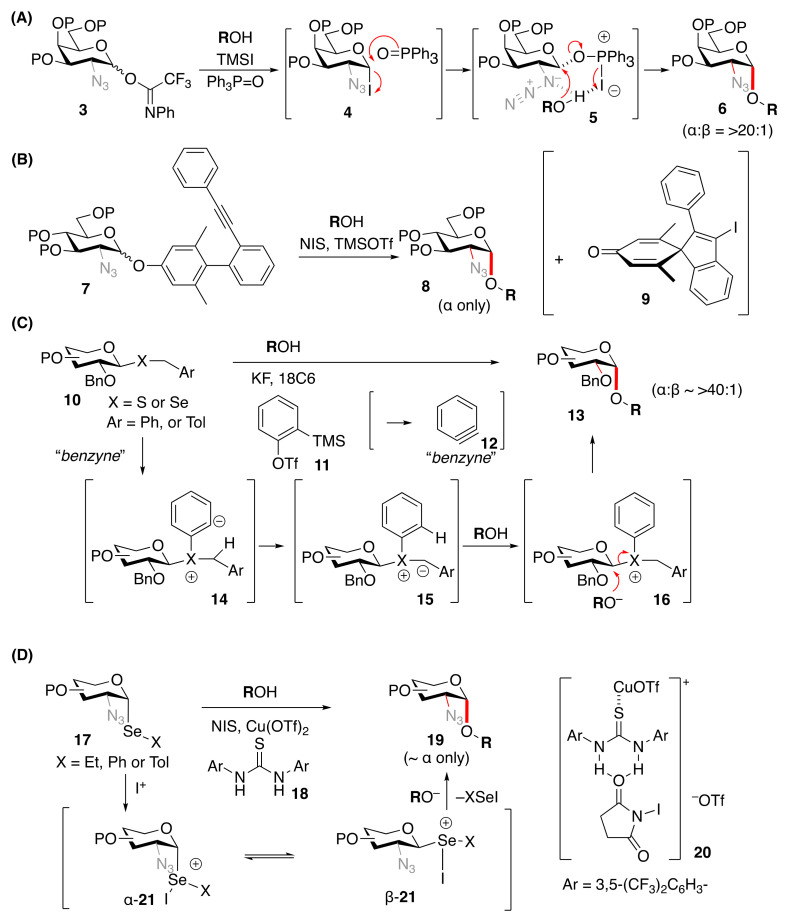

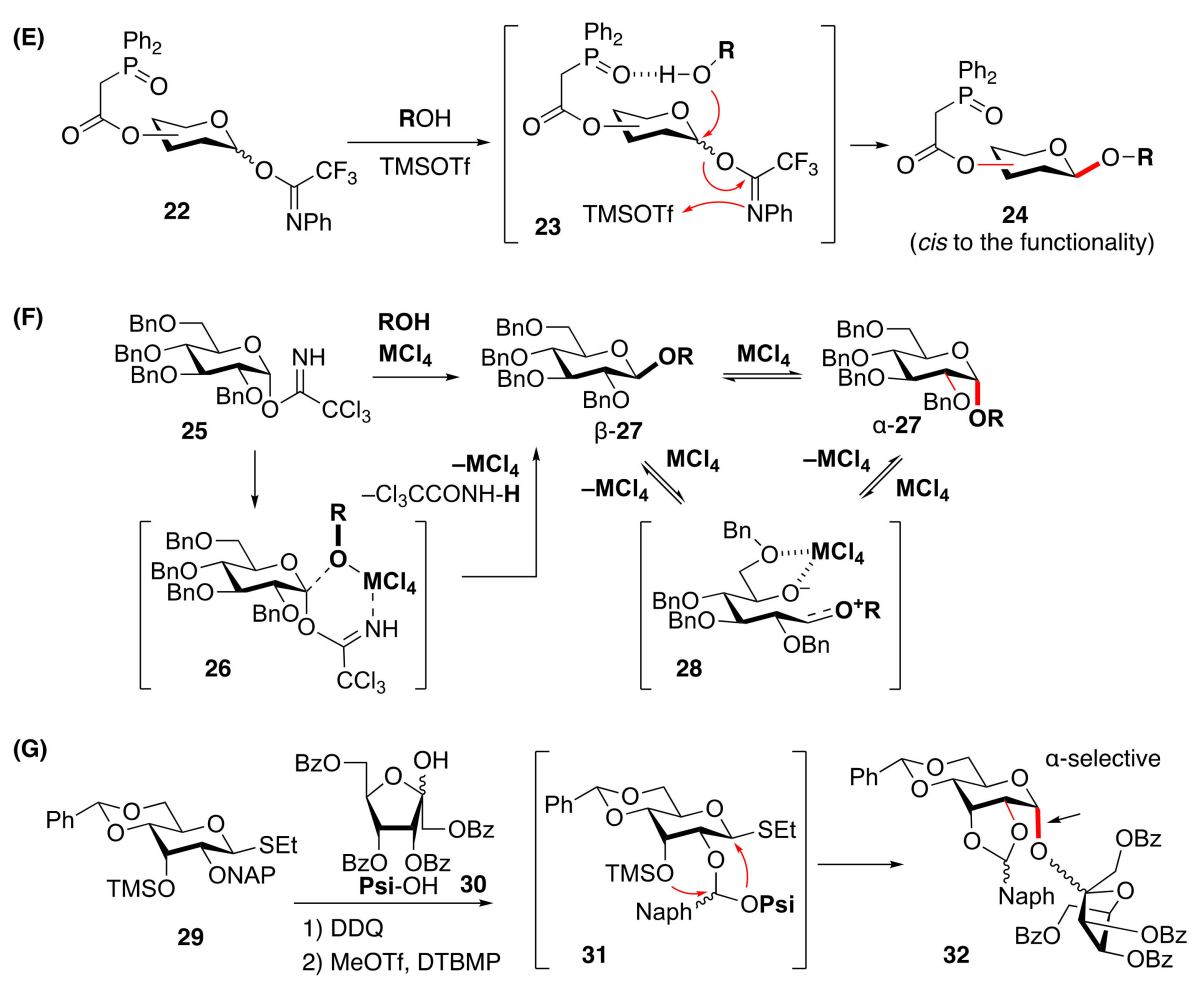
Recent progress on *cis* glycosylations. (**A**) Activation of glycosyl 2,2,2-trifluoro-*N*-phenylacetimidate by TMSI in the presence of Ph_3_P=O; (**B**) 3,5-Dimethyl-4-(2′-phenylethynylphenyl)-phenyl glycoside by NIS–TfOH; (**C**) activation of thio- and seleno-glycosides by using benzyne; (**D**) activation of seleno glycosides by using NIS–Cu(OTf)_2_–thiourea; (**E**) the remote participation of 2-(diphenylphosphinoyl)acetyl group; (**F**) SnCl_4_ or TiCl_4_–mediated stereocontrolled one-pot glycosylations. TSs (**26**, **28**) were obtained by DFT calculations (Section 2.8.3). (**G**) 2-Naphthylmethyl ether-mediated intramolecular aglycon delivery to α-D-altroside derivative with 1-OH of D-psicose acceptor. Red arrows and bonds indicate transfer of electron pairs and *cis*-linkages, respectively. Abbreviations: DDQ: 2,3-dichloro-5,6-dicyano-1,4-benzoquinone; MeOTf: methyl trifluoromethanesulfonate; DTBMP: 2,6-di-*t*-butyl-4-methylpyridine.

In recent years, further progress has been made in the development of stereoselective *O*-glycosylation as well as orthogonal techniques using various methods [42,43] as stereoselective *C*-glycosylations have been extensively developed in recent publications [44,45,46,47,48,49,50,51,52,53,54,55,56,57,58,59,60,61,62]. Glycosyl iodide as an intermediate generated from glycosyl 2,2,2-trifluoro-*N*-phenylacetimidate [CF_3_C(=NPh)–O–] (**3**) with trimethylsilyl iodide (TMSI) could be complexed with triphenylphosphine oxide (Ph_3_P=O) [63] to afford *cis* glycosides (**6**, α:β = >20:1) through the direct interaction of Ph_3_P=O with the C1 position (**4**→**5**) [64,65] (Figure 2A). Glycosyl bromide generated from thioglycoside with Br_2_ in the presence of silver trifluoromethanesulfonate (AgOTf) and 2,4,6-collidine afforded 1,2-*cis* glycoside which has been applied to a synthesis of a repeating unit of *Bacteroides fragilis* zwitterionic polysaccharide A1 [66]. Stereoselective glycosylations from 3,5-dimethyl-4-(2′-phenylethynylphenyl)phenyl glycoside (**7**) under *N*-iodosuccinimide (NIS)–trifluoromethanesulfonic acid (TfOH) conditions [67] (Figure 2B), as well as the Pd- or Cu-catalyzed activation system of donors through the cyclization of some aglycons [68,69], have been developed. For the synthesis of heparin pentasaccharide, [3+2] fragment coupling using the methodology of [67] has been applied to give the α-selective formation of the pentasaccharide (**8**). The glycosylation of **7** proceeds via an unprecedented dearomative cyclization mechanism initiated by the activation of the triple bond with I^+^, resulting in side product **9**. Benzylthio/seleno glycosides (**10**) with an activation system using benzyne (**12**), generated in situ from *o*-TMS-phenol (**11**) with trifluomethanesulphonate, KF, and 18-Crown-6 [70] (Figure 2C), have also been developed. The benzyne promotes the activation of the donor as well as the acceptor (ROH) through proposed intermediates (**14**→**16**) for an effective S_N_2 reaction. Gluco-, galacto-, and mannosyl as well as 2-deoxyglucosyl donors can be applied to afford S_N_2 products (**13**). Phenylseleno (PhSe-) 2-azidoglycosides (**17**) could be converted to 1,2-*cis* glycosides (**19**) with *N*-iodosuccinimide in the presence of a catalytic amount of Cu(OTf)_2_ and *N*,*N*-bis-[(2,4-trifluoromethyl)phenyl]thiourea (**18**) [71] (Figure 2D). The reagent combination forming the metal–organocatalyst complex (**20**) can activate selenoglycoside by the liberation of iodonium ion. The resultant α-selenonium salt analog (α-**21**) is converted to β-isomer (β-**21**), which seems to be the key intermediate for *cis* glycosylation without neighboring group participation.

Recent developments were reported about the effective participation of solvents [72,73,74,75,76,77] and additives [78,79] as well as intramolecularly participating groups [79,80,81,82,83] to induce *cis* glycosides. The participation of special functionalities such as the 2-(diphenylphosphinoyl)acetyl group (DPPA) with an acceptor has been shown to afford the *cis* glycoside (**24**) effectively through a participating intermediate (**23**) of the phosphine oxide (–Ph_2_P=O) functionality of donor (**22**) with the acceptor (ROH), as developed by Li [84,85] (Figure 2E).

By the action of M^4+^ Lewis acids such as SnCl_4_ and TiCl_4_, the glycosylation of a 2,3,4,6- tetra-*O*-benzyl-α-D-glucopyranosyl trichloroacetimidate (**25**) afforded α- and β-D-glucopyranoside (**27**) depending on the amount of M^4+^ (Figure 2F) [86]. When a catalytic amount of M^4+^ was used, β-D-glucopyranoside (β-**27**) was obtained predominantly through a proposed intermediate (**26**). The use of 3.0 equiv. of M^4+^ resulted in the formation of α-D-glucopyranoside (α-**27**) in one-pot from the donor (**25**). Since the initially obtained β-D-glucopyranoside (β-**27**) was isomerized to α-D-glucopyranoside (α-**27**) under M^4+^ conditions, the excess M^4+^ accelerated the anomerization through a proposed endo-cleavage intermediate (**28**), followed by cyclization to thermodynamically more stable α-glucoside (α-**27**). As reported by Santrsa et al., the ZnBF_4_-catalyzed glycosylation of α-imidate donor without 4,6-*O*-tethered structure in CH_2_Cl_2_ at −78 °C also afforded the β-glycoside of various donor moieties, including D-Glc*p*, and D-Gal*p*, through S_N_2 reaction without isomerization [87].

The combination of the donor, the leaving groups, and the reagent as a promotor [24,88] should be optimized. Recent progress on the orthogonal [89] one-pot procedure [90,91,92,93,94,95,96,97] using stereoselective glycosylation methods in combination with orthogonal activation systems [64,98,99,100,101,102,103,104] has afforded oligosaccharides containing 1,2-*cis* glycosidic linkages. Alternatively, 1,2-*cis* glycosylation using the naphthyl methyl ether-mediated intermolecular aglycon delivery (IAD) method [1,105,106,107,108,109,110,111,112,113,114,115,116,117] was applied to the selective 1,2-*cis* α-D-allopyranosylation using the D-allopyranosyl donor (**29**) with the 1,3,4,6-tetra-*O*-benzoyl-D-psicofuranose acceptor (**30**) through a mixed acetal intermediate (**31**) [118], which is the first example of the synthesis of non-reducing disaccharides (**32**) comprising only rare D-sugars by IAD using protected ketose (Figure 2G).

## 2. ZnI_2_-Mediated Glycosylations

**Figure 3:**
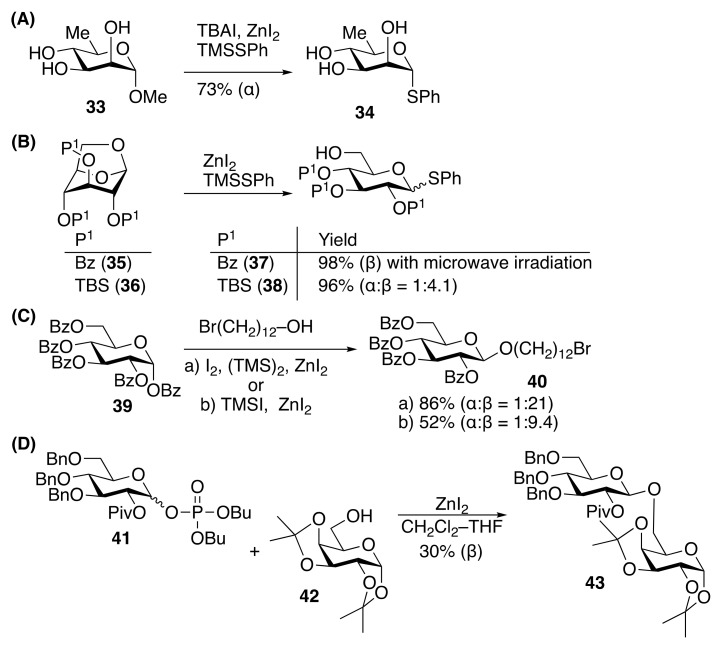
Activation of glycosyl benzoate and phosphate by ZnI_2_. (**A**) Glycosyl benzoate with TMSSR in the presence of TBAI for thioglycoside synthesis; (**B**) 1,6-Anhydroglucose derivative with TMSSR for thioglycoside synthesis; (**C**) Glycosyl benzoate with TMSI and acceptor for the synthesis of glycoside; (**D**) Glycosyl phosphate with ZnI_2_ and acceptor for the synthesis of glycoside.

Zinc iodide (ZnI_2_) has been used as a catalyst in various organic reactions [119] such as the Simmons–Smith cyclopropanation [120]. In the field of carbohydrate chemistry, both methyl glycoside and 1-*O*-benzoate have been converted to thioglycoside by the action of ZnI_2_, tetra-*n*-butyl ammonium iodide (TBAI), and alkylthiotrimethylsililane (TMSSR) [121,122,123,124], which are useful transformations for obtaining the key stable intermediate for glycosidic bond formation (Figure 3A,B). Methyl D-rhamnopyranoside (**33**) was treated with TMSSPh in the presence of ZnI_2_ and TBAI to give α-thioglycoside (**34**) in 73% [122] (Figure 3A). Benzoyl (Bz)- or tri-*t*-butyldimethylsilyl (TBS)-protected 1,6-anhydroglucose derivative (**35**, **36**) could be used as the substrate to obtain thioglycoside (**37**, **38**) by treatment under TMSSPh in the presence of ZnI_2_ without TBAI, respectively (Figure 3B).

1-*O*-Benzoate and phosphate-protected glycosyl donors (**39**, **41**) could be used for *O*-glycosylation activated by ZnI_2_ with I_2_–(TMS)_2_ [125,126] or TMSI [125,127] (Figure 3C), and ZnI_2_ [128] (Figure 3D), respectively, with glycosyl iodide as the intermediate [129]. The neighboring group participation effect of the 2-*O*-Bz group resulted in the predominant formation of 1,2-*trans* glycoside (**40**) (Figure 3C) [125]. During the screening of 1,2-*trans* glycosylation using dibutyl 2-*O*-pivaloyl-3,4,6-tri-*O*-benzyl-D-glucopyranosyl phosphate (**41**) reported by Seeberger and coworkers [128], ZnI_2_ in CH_2_Cl_2_–THF was indicated to afford β-glucoside (**43**) in 30% yield via the neighboring group participation of the 2-*O*-pivaloyl group, followed by the nucleophilic attack of an acceptor (**42**) (Figure 3D).

### 2.1. cis-Selective Glycosylations by the Action of ZnI_2_

The 1-*O*-trichloroacetimidate moiety [7,130,131,132,133] can be used as a leaving group of a glycosyl donor by the action of a cheap and mild Lewis acid, such as ZnI_2_ for *cis* glycosylation [1,89,134,135], and Zn(BF_4_)_2_ [136], B(C_6_F_5_)_3_ [137] and pyrylium salt [138] for 1,2-*trans* glycosylation. However, optimizations for the stereoselective construction of *cis* glycosides should be carried out. The conformational strain on the donor moiety caused by cyclic protective groups [139] is one of the important factors for *cis*-glycosylation [19,20,21]. There have been many recent advances in the development of ZnI_2_-mediated *O*-glycosylation reactions especially for *cis*-selective glycosylations, including α-D-glucoside, β-D-mannoside, β-D-rhamnoside, β-D-galactoside, and 2-azido-2-deoxy-α-D-glucoside formation.

### 2.2. 1,2-cis Mannosylation Using C-2-o-TsNHbenzyl Ether (TAB)

**Figure 4:**
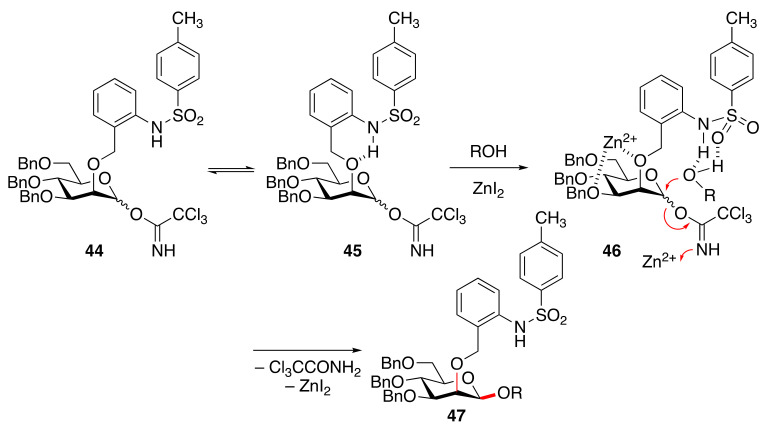
C-2-*o*-TsNHbenzyl ether (TAB)-protected mannosyl donor (**44**) under ZnI_2_ activation conditions for 1,2-*cis*-β-mannosylation.

When trichloroacetimidates are used as a convenient and common leaving group of the bimodal donor equipped with C-2-*o*-TsNHbenzyl ether (TAB) groups for gluco- [140,141], galacto- [140], and manno-sides [36], the examination of activators on the mannosylation suggested the proposed unique donor activation pathway with coordination to the donor (**44**⇄**45**) by ZnI_2_ for the stereo-direction toward 1,2-*cis* glycosidic bond formation [36] (Figure 4). Zn^2+^ not only activates the donor leaving group but also coordinates to oxygens at the 2- and 3-positions to induce the effective interaction of TAB with an incoming nucleophile during 1,2-*cis*-β-mannosylation (**44**→**47**).

### 2.3. ZnI_2_-Mediated 1,2-cis α-Glucosylation

Easily accessible and common 4,6-*O*-tethered glucosyl donors (**1^Glc^**) were found to be useful for highly stereoselective 1,2-*cis* α-glucosylation mediated by ZnI_2_ [37] (Figure 1). The 4,6-*O*-tethering constrains a pyranose ring of the glycosyl donors for stereoselective 1,2-*cis* glycosylation [19]. The versatility and effectiveness of the α-glucosylation strategy was demonstrated successfully with various acceptors. This approach demonstrates the feasibility of the modular synthesis of α-glucans with both linear and branched backbone structures. DFT calculations (vide infra) indicated that both the activation of trichloroacetimidate and the coordination between 2–O in the donor moiety and the hydroxy group in the acceptor could be carried out by Zn^2+^, and that 1,2-*cis* selective glycosylation proceeded through the proposed transition state (TS) structure (**Glc TS**) after activation to afford α-glucoside (α-**2^Glc^**).

### 2.4. ZnI_2_-Mediated 1,2-cis β-D-Mannopyranosylation and β-L-Rhamnopyranosylation

**Figure 5:**
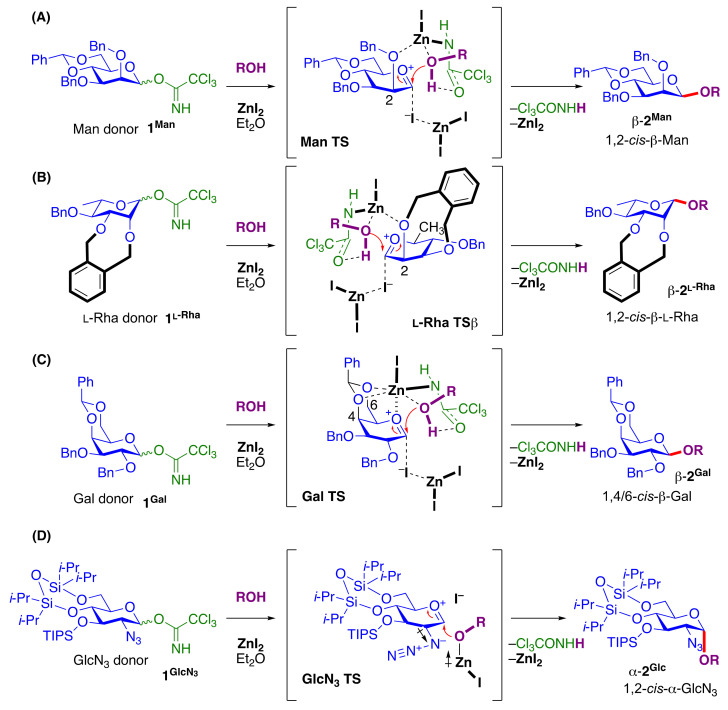
ZnI_2_–mediated *cis*-glycosylations. (**A**) 1,2-*cis* β-D-mannopyranosylation; (**B**) 1,2-*cis* β-L-rhamnopyranosylation; (**C**) 1,4/6-*cis* β-D-galactopyranosylation; (**D**) 1,2-*cis* 2-azido-2-deoxy-α-D-glucopyranosylation. TSs were obtained by DFT calculations (Section 2.8.1 and Section 2.8.2).

The ZnI_2_-mediated method could be applied to the synthesis of 1,2-*cis* β-glycosides such as β-D-mannopyranosides (β-**2^Man^**) [38] (Figure 5A) and β-L-rhamnopyranosides (β-**2^L-Rha^**) [39] which are 6-deoxy-β-L-mannopyranosides [142] (Figure 5B). The 1,2-*cis* β-manno- and β-L-rhamno-sylation mediated by ZnI_2_ employed easily accessible 4,6-*O*-tethered mannosyl and L-rhamnosyl trichloroacetimidate donors (**1^Man^** and **1^L-Rha^**). The versatility and effectiveness of this strategy were demonstrated with successful β-mannosylation of a wide variety of alcohol acceptors, including complex natural products, amino acids, and glycosides.

Through iterative ZnI_2_-mediated mannosylation with a chitobiosyl azide acceptor, followed by the site-selective deprotection of the mannosylation product, this novel methodology enables the modular synthesis of a key intermediate trisaccharide with a β-D-Man-(1→4)-β-D-GlcNAc-(1→4)-β-D-GlcNAc linkage for *N*-glycan synthesis [38]. The core repeating tetrasaccharide unit with an α-L-Rha*p*-(1→2)-β-D-Gal*p*-(1→4)-β-L-Rha*p*-(1→4)-α-D-Glc*p* linkage of the *Streptococcus pneumoniae* 23F capsule polysaccharide has been successfully synthesized using ZnI_2_-mediated 1,2-*cis* β-L-rhamnosylation with a convergent [2 + 2] strategy [39].

DFT calculations also suggested similar activation and coordination via the key coordinated-intermediates (**Man TS** and **L-Rha TS**) in the aforementioned α-glucosylation. Theoretical investigations using DFT calculations (vide infra) delved into the mechanistic details of this β-selective glycosylation and elucidated the essential roles of two zinc cations as the activating agent of the donor and the principal mediator of the *cis*-directing intermolecular interaction [38,39].

### 2.5. ZnI_2_-Mediated 1,4/6-cis β-D-Galactopyranosylation

Although the ZnI_2_-mediated method has been applied to the synthesis of α-D-galactopyranosides, the β-anomer (β-**2^Gal^**) was obtained from the 4,6-*O*-tethered 2,3-di-*O*-benzyl-D-galactopyranosyl trichloroacetimidate donor (**1^Gal^**) in the presence of ZnI_2_ in a 1,2-*trans* glycosylation fashion [40] (Figure 5C). The unexpected formation of β-D-galactopyranosides (β-**2^Gal^**) [143] could be explained by the favored coordination of Zn^2+^ to the conformationally fixed 4-*O* or 6-*O* of the galactopyranosyl donor instead of 2-*O* as in the expected cases. Suggested by DFT calculations (vide infra), the stability of the key intermediate for the stereodirection was proposed to be enhanced by the coordination of Zn^2+^ to 4-*O* or 6-*O* on the β-face that controlled the approach of the acceptor from the β-side. This β-D-galactopyranosylation should occur through 1,4/6-*cis* glycosylation under ZnI_2_ conditions via **Gal TS**. By using this ZnI_2_-mediated β-galactosylation strategy, the tetrasaccharide fragment β-D-Gal*p*-(1→6)-3-*O*-[α-L-Ara*f*-(1→)]-β-D-Gal*p*-(1→6)-β-D-Gal*p* linkage of arabinogalactan, derived from the plant polysaccharide of S*tevia rebaudiana* and *Silybum marianum*, was synthesized efficiently with high stereoselectivity [40].

### 2.6. ZnI_2_-Mediated 1,2-cis 2-azido-2-deoxy-α-D-Glucopyranosylation

The synthesis of the 1,2-*cis* 2-acetamido-2-deoxyglucoside (D-GlcNAc) core of the capsular polysaccharide (CPS) remains challenging. The tetraisopropyldisiloxane (TIPDS)-protected 2-azido-2-deoxy-D-glucosyl donor (**1^GlcN^^3^**) afforded the α-glycoside (**2^GlcN^^3^**) (α:β = >20:1) predominantly in maximum yield [41] (Figure 5D). This approach applies to a wide acceptor substrate scope, including various aliphatic alcohols, sugar alcohols, and natural products. The reaction mechanism was explored by combined experimental variable-temperature NMR (VT-NMR) studies, mass spectrometry (MS) analysis, and DFT calculations (vide infra), and the results suggested the formation of a covalent α-C1^GlcN3^-iodide intermediate in equilibrium with a separated oxocarbenium–counter ion pair, followed by an S_N_1-like α-nucleophilic attack most likely from the separated ion pairs by the ZnI_2_-activated acceptor complex under the influence of the 2-azido gauche effect [144] via **GlcN_3_ TS**. The α-D-GlcNAc-linked core structure of the CPS repeating fragments from *Acinetobacter baumannii* was synthesized by employing the developed reaction as the key step for constructing the 1,2-*cis* 2-azido-2-deoxy glycosidic linkage.

### 2.7. ZnI_2_-Mediated 1,2-cis β-D-Arabinofuranosylation

**Figure 6:**
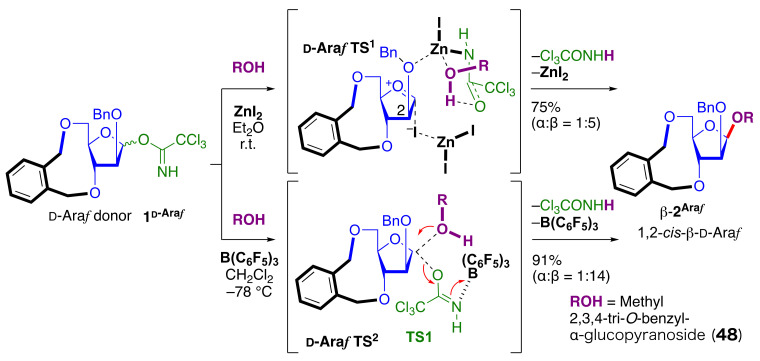
B(C_6_F_5_)_3_–promoted 1,2-*cis* D-arabinofuranosylations of 3.5-*O*-xylylene protected donor. TS was obtained by DFT calculations (Section 2.8.2).

In the case of D-arabinofuranosylation using a D-arabinofuranosyl trichloroacetimidate as the donor, tris(pentafluorophenyl)borane [B(C_6_F_5_)_3_] conditions resulted in better 1,2-*cis* stereoselectivity compared to the ZnI_2_-mediated reaction [145] (Figure 6). The reaction of D-arabinofuranosyl trichloroacetimidate (**1D-Ara*f***) under ZnI_2_ activation conditions proceeded 1,2-*cis* stereoselectively via the proposed TS structure (**D-Ara*f* TS1**) when the donor was protected as an 8-membered 3,5-*O*-xylylene group [146,147,148] such as the 9-membered 3,5-*O*-tetra-*i*-propyldisiloxanylidene (TIPDS) group [149,150,151]. Since the 3,5-*O*-xylylene-protected donor gave higher yield, the optimization of the conditions including the promotor suggested that B(C_6_F_5_)_3_ [142,152,153,154,155] could activate the α-imidate and directly afford the glycosidic bond through an S_N_2-like mechanism via **D-Ara*f* TS2** from α-D-arabinofuranosyl trichloroacetimidate at −78 °C in CH_2_Cl_2_. This method was applied to the synthesis of β-D-Ara*f* linkages with various acceptors including the acceptor for producing a non-reducing terminal structure of mycobacterial arabinan fragment (**2D-Ara*f***) [145,156].

### 2.8. Density Functional Theory (DFT) Calculations for ZnI_2_-Mediated Reactions

#### 2.8.1. DFT Calculations for ZnI_2_-Mediated Glucosylation and Mannosylation

The TSs of each ZnI_2_-mediated reaction were obtained by density functional theory (DFT) computations with Gaussian 16 software package [157] and the detailed conditions are as follows. For **GlcTS** (Figure 1) and **ManTS** (Figure 2A), structures of plausible reagents, products, and intermediates species were preoptimized at the PM6-d3 level of theory [158,159] at the gas phase, and then subjected to geometry optimization at the PBE0/def2-TZVP level of theory [160,161,162], with Grimme’s DFT-D3(BJ) empirical dispersion correction [163] applied to account for the dispersion interactions, and the implicit solvation model based on density (SMD) [164] applied to describe the solvent effect exerted by diethyl ether. A short intrinsic reaction coordinate scan with the local quadratic approximation [165] method and 0.1 Bohr step-size was performed on the optimized TS structures at the same level of theory for 30 steps on each side to ensure that the TS structures correspond to energy maxima along the reaction routes connecting the immediately reacting intermediates and product structures. The accurate electronic energy of the chemical species was calculated from the optimized structures with single-point calculation at the M06-2X/ma-def2-TZVPP/SMD (solvent = diethyl ether) level of theory [166,167,168] with an ultrafine integration grid, and the thermal energy terms associated with various thermal motions of the molecule as well as the solvation of the molecules in diethyl solvent were calculated from the frequency analysis output using Shermo [169], with scale factors [170] applied to adjust for the errors arising from the harmonic oscillation assumption. The Gibbs free energy of each chemical species was calculated as the sum of the accurate electronic energy and thermal energy terms.

#### 2.8.2. DFT Calculations for ZnI_2_-Mediated l-Rhamnosylation, 2-azido-2-deoxy-α-d-Glucopyranosylation and for B(C_6_F_5_)_3_-Mediated d-Arabinofuranosylation

For **L-Rha TS**, **GlcN_3_ TS** (Figure 5B,D) and **D-Ara*f* TS^2^** (Figure 6), the geometries were optimized at the theory level of B3LYP [171,172]//BS1 (BS1 = 6-31G(d) [173,174] for main group elements and Lanl2dz [175] for Zn, Si, I, and Sn) in the gas phase. Solvation free energies were calculated using the SMD [164] solvation model (solvent = diethyl ether or DCM) under ωB97XD [176] or M06 [177]//BS2 (BS2 = 6-311 + G** [173,174] for main group elements and SDD [178] for Zn, Si, I, and Sn). The Gibbs free energy present in this paper is the sum of single-point energy at ωB97XD [173] or M06 [174] //BS2, thermodynamic correction at B3LYP//BS1, and solvation free energy.

#### 2.8.3. DFT Calculations for ZnI_2_-Mediated D-Galactopyranosylation

For **Gal TS** (Figure 5C), DFT computations at the TPSS/def-TZVP/SMD and diethyl ether [173,174,175] level of theory, with Grimme’s empirical correction to account for dispersion D3(BJ) [163], were carried out.

## 3. Conclusions

For a 1,2-*trans* selective glycosylation, methodologies have been further developed using 2-(2-propylsulfinyl)benzyl 1,2-orthoester glycosides [179] activated by trifluoromethanesulfonic anhydride (Tf_2_O) with DTBMP, *N*-(1,1-dimethylpropargyl)carbamate by chloro[tris(2,4-di-*t*-butyphenyl)-phosphite]gold and AgOTf [180,181,182] and diphenyl phosphate by bis-thiourea type molecule catalyst with high site-selection [183].

There have been extremely valuable reports on the synthesis of large glycans with over 20 monosaccharide units [184] composed of pyranosides [185,186,187,188,189,190,191,192], furanosides [193,194,195,196,197], and both isomers [198]. Both the synthesis and the application of biosynthetic incorporation and selective labeling [199,200,201] of mycobacterial cell walls [202,203] related to tuberculosis [204,205] should be noted as recent advances in remodeling glycoconjugates containing *cis* glycosides.

This review summarizes recent stereoselective glycosylation methods including the very simple and efficient ZnI_2_-mediated *cis*-glycosylation methodology using constrained glycosyl trichloroacetimidate donors such as D-Glc*p*, D-Man*p*, L-Rha*p*, and 2-azido-2-deoxy-D-Glc*p* in a 1,2-*cis* selective manner, and D-Gal*p* in a 1,4/6-*cis* selective manner. In addition, other findings for β-D-Ara*f* formation using a constrained donor in the presence of B(C_6_F_5_)_3_ instead of ZnI_2_ as well as the synthesis of branched terminal D-arabinan hexasaccharide fragment using tricloroacetimidate donor-B(C_6_F_5_)_3_ combination were also effective enough. These methodologies, mainly based on ZnI_2_-mediated *cis*-glycosylation with optimization, could be used in investigations focused on elucidating the biosynthetic pathways and function of these glycans, and conjugating them to a protein carrier for vaccine generation in the case of antigenic glycans from pathogenic bacteria. Further exploration of applications of this methodology for the synthesis of other complex oligosaccharides containing *cis* linkages is the focus of continuing investigation for synthetic carbohydrate chemists.

## Data Availability

No new data were created or analyzed in this study. Data sharing is not applicable to this article.
